# Fewer screens, greater needs: housing insecurity and healthcare costs for transgender patients in a safety-net system

**DOI:** 10.1093/haschl/qxaf226

**Published:** 2025-12-05

**Authors:** Aaron S Breslow, Gray Babbs, Elizabeth Cavic, Iby Thomas, Isabella Gibaldi, Ana M Progovac, Arjee Restar, Ginette M Sims, Jonathan Alpert, Benjamin Lê Cook, Kevin P Fiori, Samantha Levano, Earle C Chambers

**Affiliations:** PRIME Center for Health Equity, Department of Psychiatry and Behavioral Sciences, Albert Einstein College of Medicine, Bronx, NY 10461, United States; Department of Health Services, Policy, and Practice, Brown University School of Public Health, Providence, RI 02903, United States; PRIME Center for Health Equity, Department of Psychiatry and Behavioral Sciences, Albert Einstein College of Medicine, Bronx, NY 10461, United States; Department of Family and Social Medicine, Albert Einstein College of Medicine, Bronx, NY 10461, United States; Albert Einstein College of Medicine, Bronx, NY 10461, United States; Department of Population Health, NYU Grossman School of Medicine, New York, NY 10003, United States; Health Evaluation Research Lab, Department of Psychiatry, Cambridge Health Alliance, Harvard Medical School, Boston, MA 02141, United States; Departments of Epidemiology, and Health Systems and Population Health, University of Washington School of Public Health, Seattle, WA 98195, United States; School of Public Health, Yale University, New Haven, CT 06510, United States; Weitzman Institute, Moses Weitzman Health System, Washington, DC 20005, United States; PRIME Center for Health Equity, Department of Psychiatry and Behavioral Sciences, Albert Einstein College of Medicine, Bronx, NY 10461, United States; PRIME Center for Health Equity, Department of Psychiatry and Behavioral Sciences, Albert Einstein College of Medicine, Bronx, NY 10461, United States; PRIME Center for Health Equity, Department of Psychiatry and Behavioral Sciences, Albert Einstein College of Medicine, Bronx, NY 10461, United States; Health Evaluation Research Lab, Department of Psychiatry, Cambridge Health Alliance, Harvard Medical School, Boston, MA 02141, United States; Department of Family and Social Medicine, Albert Einstein College of Medicine, Bronx, NY 10461, United States; Department of Family and Social Medicine, Albert Einstein College of Medicine, Bronx, NY 10461, United States; PRIME Center for Health Equity, Department of Psychiatry and Behavioral Sciences, Albert Einstein College of Medicine, Bronx, NY 10461, United States; Department of Family and Social Medicine, Albert Einstein College of Medicine, Bronx, NY 10461, United States

**Keywords:** transgender, disparities, health-related social needs, housing, healthcare costs, safety-net healthcare system

## Abstract

**Introduction:**

Routine screening for health-related social needs (HRSNs) is inconsistent, creating disparities in who gets identified and supported. Transgender patients, already facing structural stigma, may be especially affected.

**Methods:**

We analyzed electronic health records from a large urban safety-net system (2018-2023). We identified 2639 transgender patients with at least one outpatient visit and created a ∼11:1 comparison cohort of 23 944 cisgender patients. Overall, 7.5% (*n* = 1997) completed a Social Needs Questionnaire (SNQ), including 1840 cisgender and 157 transgender patients. We compared screening rates using chi-square tests and assessed adjusted odds for HRSN with logistic regression.

**Results:**

Transgender patients were screened less often than cisgender patients (5.9% vs 7.7%, *P* = 0.001). Among those screened, they had more than twice the odds of housing instability, poor-quality housing, and healthcare costs. Odds for interpersonal violence were th3ree times higher. Findings were consistent in sensitivity analyses adjusting for age, insurance, and neighborhood.

**Conclusion:**

Transgender patients were underscreened yet faced greater HRSNs. Standardized screening and expanded supports are critical to support transgender communities.

Transgender and nonbinary people in the United States face pervasive structural stigma that directly impacts their health outcomes.^1-3^ Structural stigma includes systemic barriers such as discriminatory laws, healthcare policies, and societal norms that limit access to critical resources. These barriers contribute to socioeconomic disparities, including reduced formal employment opportunities and income instability, which can compound barriers to healthcare access.^4,5^ A recent nationally representative study found that transgender adults were significantly more likely than cisgender adults to experience job loss (22.1% vs 12.2%), inability to pay housing bills (21% vs 11.6%), and transportation barriers (21.2% vs 8%).^6^ These inequalities shape long-term health trajectories and limit access to essential medical care.^7,8,9^

In addition to structural stigma, transgender people face pervasive interpersonal stigma, including discrimination from healthcare providers, family members, and peers.^1-3^ Transgender people are ∼4 times as likely as cisgender people to experience violent victimization, including sexual assault and physical harassment.^10,11^ These combined effects of structural and interpersonal stigma increase stress-related health disparities,^12-15^ including elevated rates of depression, anxiety, and suicidality, and chronic conditions such as hypertension, kidney disease, and congestive heart failure.^16,17^ Additionally, a 2020 survey found that two thirds of transgender respondents reported experiencing healthcare discrimination within the past year.^18^ This discrimination, whether overt or subtle, often leads transgender people to delay or avoid seeking healthcare, increasing their risk of untreated health conditions and emergency health concerns. Around 22% of transgender people report avoiding healthcare due to anticipated discrimination,^19,20^ while many report delaying care to the extent that it results in medical emergency.^21^ This avoidance of healthcare is compounded by financial barriers to care.

Health-related social needs (HRSNs), defined as an individual's unmet material conditions, are critical determinants of health outcomes and psychological well-being.^22^ Housing insecurity, food insecurity, interpersonal stressors, and healthcare affordability are examples of HRSNs that yield direct and indirect associations with long-term health outcomes. Addressing HRSNs in clinical settings through routine screening and referrals may help mitigate health disparities,^23^ yet little is known about the successes of this type of measurement-based care among transgender patients in large healthcare systems.^24^

Despite growing recognition of transgender health inequity, most research on HRSNs, among transgender people in particular and in the general population, has relied on survey-based data rather than clinical implementation studies.^24,25^ While surveys are critical for measuring unmet needs, fewer studies have evaluated how health systems may document, intervene on, or even reduce HRSNs in real-world clinical settings.^26^As noted in recent reviews, this represents a breakdown in the HRSN care continuum: from screening to referral to outcomes, where gaps in implementation and follow-through may buffer clinical impact.^27^ This disconnect is especially concerning for transgender people,^6^ whose material needs often go unscreened and unmet in traditional care settings.^28^

This study thus aims to contribute to this growing evidence base by addressing 2 objectives: (1) to examine disparities in the documentation of HRSNs between transgender and cisgender patients and (2) to compare the odds for HRSNs in these groups. By addressing these questions, we aim to inform interventions that promote health equity for transgender people.

## Materials and methods

### Setting and patients

This study utilized data from Montefiore Medical Center, the largest integrated safety-net healthcare system in the Bronx, NY. Montefiore serves a predominantly low-income, racially, and ethnically minoritized population through its network of 14 hospitals and over 200 outpatient sites. The study included adults aged 18-65 with at least one outpatient visit between April 2018 and June 2023, when the healthcare system began to administer the Social Needs Questionnaire (SNQ) tailored for its patient population.

Transgender patients were identified using a validated 3-step method harnessing secondary electronic health record (EHR) data:^29,30^ (1) individuals with International Classification of Disease-Clinical Modification (ICD-9-10-CM) codes for gender dysphoria or related concerns; (2) individuals with a transgender identity documented in the sexual orientation and gender identity (SOGI) SmartForm, a tool embedded within the EHR; and (3) individuals with documented changes to their recorded sex attributed to “sex reassignment.” Structured SOGI fields were added to the EHR in 2016 as part of a system-wide equity initiative, driven by participation in the Human Rights Campaign's Healthcare Equality Index, which incentivized SOGI data collection.^31^ To create a comparison cohort, we randomly selected an 11:1 sample of cisgender patients from the healthcare system's EHR who did not meet any of the transgender inclusion criteria. Both groups included patients with at least one outpatient visit during the study period.

### Measures

#### Health-related social needs

HRSNs were assessed using the SNQ, a 10-item survey adapted from the validated Health Leads Social Needs Screening Toolkit and integrated into Montefiore's EHR in April 2018.^24,32^ Health Services, Policy, and Practice, Brown University School of Public Health, Providence, RI 02903. Patients were asked about housing instability, poor-quality housing, utility shutoffs, food insecurity, transportation challenges, healthcare cost burdens, child or elder care needs, legal issues, interpersonal stress, and interpersonal violence. While the SNQ was available for use across outpatient sites, providers determined which patients to screen based on site capacity and clinical discretion. Among patients who completed the SNQ multiple times, we retained the highest reported need for each item. Providers reviewed results with patients and offered referrals to community health workers and social workers to provide navigation assistance.^23,27^

#### Demographic characteristics

Demographic data included race and ethnicity, insurance, and neighborhood characteristics. These data were extracted from the EHR and may reflect either self-report at registration or staff-entered administrative records. Because not all entries are self-reported, race and ethnicity may be inconsistently captured and should be interpreted with caution.

Insurance types included private insurance, Medicare, Medicaid, no insurance, and missing data. Neighborhood characteristics were determined using ZIP code–linked American Community Survey data (to access the appendix, please refer to the Supplementary material online).^33^ Neighborhoods were classified as low income if ≥20% of residents lived below the federal poverty line, low education if ≥25% of adults had less than a high school diploma, and high unemployment if ≥10% of the labor force was unemployed.

### Statistical analyses

First, we used chi-square tests (α = 0.05) to compare HRSN documentation rates between transgender and cisgender patients and across other sociodemographic characteristics. Second, among patients with documented screenings, we ran age-adjusted logistic regression models to estimate adjusted odds ratios (aORs) for each HRSN, with cisgender patients as the reference group. The Benjamini–Hochberg (B–H) correction was applied to control the false discovery rate across multiple comparisons; results were considered significant when the *P*-value was below the corresponding B–H critical value.

Third, in sensitivity analyses, we ran models additionally adjusting for insurance status and neighborhood socioeconomic indicators.^33^ Because these factors reflect downstream structural determinants rather than confounders, we treated them as mediators. Age-adjusted results are presented in the main text; fully adjusted models appear in Table A2 (to access the appendix, please refer to the Supplementary material online).

We could not model clinic- or provider-level variation in gender identity documentation or SNQ administration because site identifiers were inconsistently formatted across the 200+ outpatient sites.^23,34^

## Results

Following the 3-step identification method, we identified a cohort of 2898 likely transgender adult patients with at least one outpatient visit between April 2018 and July 2023. Among them, 79.8% were identified via ICD-9-10-CM codes, 48.0% via a transgender identity on their SOGI SmartForm, and 14.7% via a recorded “sex reassignment” in their chart. To create a ∼11:1 comparison cohort, we randomly selected 23 944 cisgender patients from the EHR database. This led to a final sample of 26 583 patients with at least one outpatient visit during the study period.

### Differences in screening

Among the 26 583 patients included in the study, we observed group differences in documented HRSN screening (Table 1). Transgender patients were significantly less likely than cisgender patients to have documented SNQ completion (5.9% vs 7.7%, *P* = 0.001). Cisgender women had the highest completion rate (9.0%) compared with cisgender men (6.0%, *P* < 0.001). Among transgender subgroups, trans men (6.4%) and nonbinary individuals (6.1%) had slightly higher rates than transgender women (5.5%, *P* = 0.001).

**Table 1.** Demographics of patients with at least one visit from April 2018 (total *N* = 26 583) who did not complete the SNQ compared with those who did complete the SNQ.

	Completed the SNQ?		
	No (*n* = 24 586)	Yes (*n* = 1997)	*P*-value
Age, mean (SD)	40.7 (14.1)	40.8 (14.0)	0.9
	*n* (%)	*n* (%)	
Transgender	2482 (94.1%)	157 (5.9%)	
Cisgender	22 104 (92.32%)	1840 (7.68%)	
Gender identity			<0.001
Nonbinary	262 (93.9%)	17 (6.1%)	
Trans woman	587 (94.5%)	34 (5.5%)	
Trans man	407 (93.6%)	28 (6.4%)	
Cis woman	12 433 (91%)	1223 (9%)	
Cis man	9640 (94%)	617 (6%)	
Missing	1257 (94.2%)	78 (5.8%)	
Race and ethnicity			<0.001
Hispanic/Spanish/Latino	5574 (87.9%)	764 (12.1%)	
Non-Hispanic American Indian/Alaskan Native	68 (84%)	13 (16%)	
Non-Hispanic Asian/Pacific Islander	598 (90.6%)	62 (9.4%)	
Non-Hispanic Black	4787 (88.5%)	625 (11.5%)	
Non-Hispanic White	5021 (96.4%)	185 (3.6%)	
Another race that is not listed	2697 (95.3%)	134 (4.7%)	
Patient unavailable or declined to report	5841 (96.5%)	214 (3.5%)	
Lifetime insurance			<0.001
Private insurance	9573 (91.8%)	853 (8.2%)	
Medicare	970 (88.5%)	126 (11.5%)	
Medicaid	9067 (90%)	1002 (10%)	
Uninsured	36 (97.3%)	1 (2.7%)	
Missing	4940 (99.7%)	15 (0.3%)	
Low-income neighborhood	8790 (36.1%)	961 (48.1%)	<0.001
Low-education neighborhood	7030 (28.9%)	832 (41.7%)	<0.001
High-unemployment neighborhood	7356 (30.2%)	863 (43.2%)	<0.001

Displays the demographic characteristics of the patients who did not complete the SNQ (*n* = 24 586) compared with those who did complete the SNQ (*n* = 1997), as well as *P*-value for omnibus chi-square tests of significant difference at the α = 0.05 level. Source: Analysis of Montefiore's electronic health record data, 2018-2023.

Abbreviations: SD, standard deviation; SNQ, Social Needs Questionnaire.

Significant differences in SNQ completion were observed by race and ethnicity. Hispanic (12.1%) and non-Hispanic Black patients (11.5%) were most likely to have documented SNQ completion, while non-Hispanic White patients (3.6%) and those who declined to report race (3.5%) had the lowest rates (*P* < 0.001). Patients with Medicaid (10.0%) and Medicare (11.5%) were more likely to complete the SNQ compared with those with private insurance (8.2%) or no insurance (2.7%, *P* < 0.001).

Screening rates also differed by neighborhood. Patients in low-income areas were screened more often than those in higher-income areas (48.1% vs 36.1%, *P* < 0.001), and screening was similarly higher in neighborhoods with low educational attainment (41.7% vs 28.9%, *P* < 0.001) and high unemployment (43.2% vs 30.2%, *P* < 0.001).

### Demographics of patients with documented HRSNs

Among the 1997 patients who completed the SNQ, we also found demographic differences between the cisgender (*n* = 1840) and transgender (*n* = 157) groups (Table 2). Significant differences were observed between the 2 groups in age, gender identity, race/ethnicity, and insurance coverage. Transgender patients were significantly younger on average than cisgender patients (mean age 32.3 years vs 41.4 years, *P* < 0.001). Gender identity distributions also differed markedly between the groups (*P* < 0.001). Among cisgender patients, most were cisgender women (66.5%), followed by cisgender men (33.5%). In contrast, transgender patients included trans women (21.7%), trans men (17.8%), and nonbinary individuals (10.8%), with 49.7% missing detailed gender identity data.

**Table 2.** Demographic characteristics among patients who completed the SNQ (total *N* = 1997), compared between cisgender and transgender patients.

	Cisgender	Transgender	*P*-value
	(*n* = 1840)	(*n* = 157)	
Age, mean (SD)	41.4 (13.9)	32.3 (12.7)	<0.001
	*n* (%)	*n* (%)	
Gender identity			<0.001
Nonbinary	0 (0.0%)	17 (10.8%)	
Trans woman	0 (0.0%)	34 (21.7%)	
Trans man	0 (0.0%)	28 (17.8%)	
Cis woman	1223 (66.5%)	0 (0.0%)	
Cis man	617 (33.5%)	0 (0.0%)	
Missing	0 (0.0%)	78 (100.0%)	
Race and ethnicity			0.034
Hispanic/Spanish/Latino	716 (38.9%)	48 (30.6%)	
Non-Hispanic American Indian/Alaskan Native	12 (0.7%)	<11	
Non-Hispanic Asian/Pacific Islander	58 (3.2%)	<11	
Non-Hispanic Black	574 (31.2%)	51 (32.5%)	
Non-Hispanic White	174 (9.5%)	11 (7.0%)	
Another race that is not listed	115 (6.2%)	19 (12.1%)	
Patient unavailable or declined to report	191 (10.4%)	23 (14.6%)	
Lifetime insurance			<0.001
Private insurance	800 (44.1%)	41 (26.1%)	
Medicare	119 (6.1%)	<11	
Medicaid	908 (49.0%)	103 (65.0%)	
Uninsured	<11	<11	
Missing	12 (0.7%)	<11	
Low-income neighborhood	870 (47.4%)	85 (54.8%)	0.076
Low-education neighborhood	764 (41.7%)	72 (46.5%)	0.24
High-unemployment neighborhood	807 (43.1%)	71 (47.1%)	0.32

Displays the demographic characteristics among patients who completed the SNQ, with row percentages displayed among the cisgender and transgender cohorts, as well as *P*-value for omnibus chi-square tests of significant difference between cohorts at the α=0.05 level. Source: Analysis of Montefiore's electronic health record data, 2018-2023.

Abbreviation: SD, standard deviation; SNQ, Social Needs Questionnaire.

Race and ethnicity distributions showed notable differences between cisgender and transgender patients (*P* = 0.034). Non-Hispanic Black patients represented a similar proportion in both groups (31.2% of cisgender patients and 32.5% of transgender patients). Hispanic/Latino patients were more prevalent among cisgender patients (38.9%) compared with transgender patients (30.6%), while patients identifying as “another race not listed” were more common among transgender patients (12.1% vs 6.2%). Additionally, 14.6% of transgender patients declined to report their race/ethnicity compared with 10.4% of cisgender patients.

Significant disparities were observed in insurance status between the 2 groups (*P* < 0.001). Transgender patients relied on Medicaid more than cisgender patients (65% vs 49%), while cisgender patients had higher rates of private insurance (44.1% vs 26.1%). Transgender patients had fewer instances of Medicare coverage, but these numbers were small.

While transgender patients were more likely to reside in low-income neighborhoods (54.8% vs 47.4%), low-education neighborhoods (46.5% vs 41.7%), and high-unemployment neighborhoods (47.1% vs 43.1%), these differences were not statistically significant.

### Differences in HRSNs

Table 3 summarizes the unadjusted prevalence of HRSNs among cisgender (*n* = 1840) and transgender (*n* = 157) patients who completed the SNQ. Key findings highlight significant disparities in several social needs, with transgender patients consistently reporting significantly higher burden of housing instability, poor-quality housing, healthcare costs, and interpersonal violence compared with cisgender patients.

**Table 3.** Unadjusted prevalence, absolute difference, and relative difference of each health-related social need among patients who completed the SNQ (*n* = 1997).

Social need	Unadjusted prevalence			Absolute difference (pp)	Relative difference	*P*-value
	Cisgender	Transgender	Total			
	(*n* = 1840)	(*n* = 157)	(*n* = 1997)			
1. Housing instability: “Are you worried that in the next 2 months, you may not have a safe or stable place to live? (eviction, being kicked out, homelessness)?”	6.30%	11.46%	6.71%	5.16	81.9%	0.013
2. Poor-quality housing: “Are you worried that the place you are living now is making you sick? (has mold, bugs/rodents, water leaks, not enough heat)?”	5.65%	11.46%	6.11%	5.81	102.8%	0.004
3. Utilities shut off: “In the last 3 months, has the electric, gas, or water company threatened to shut off services to your home?”	4.13%	5.73%	4.26%	1.60	38.7%	0.34
4. Food insecurity: “In the last 12 months, did you worry that your food could run out before you got money to buy more?”	7.39%	10.83%	7.66%	3.44	46.5%	0.12
5. Healthcare transportation: “In the last 3 months, has lack of transportation kept you from medical appointments or getting your medications?”	5.87%	8.28%	6.06%	2.41	41.1%	0.224
6. Healthcare costs: “In the last 3 months, did you have to skip buying medications or going to doctor's appointments to save money?”	5.43%	12.10%	5.96%	6.67	122.8%	0.001
7. Child or elder care: “Do you need help getting child care or care for an elderly or sick adult?”	2.55%	3.18%	2.60%	0.63	24.7%	0.634
8. Legal help: “Do you need legal help? (child/family services, immigration, housing discrimination, domestic issues, etc?)”	3.26%	5.73%	3.46%	2.47	75.8%	0.104
9. Interpersonal stress: “Are you finding it so hard to get along with a partner, spouse, or family members that it is causing you stress?”	3.86%	5.10%	3.96%	1.24	32.1%	0.445
10. Interpersonal violence: “Does anyone in your life hurt you, threaten you, frighten you or make you feel unsafe?”	0.98%	3.18%	1.15%	2.20	224.5%	0.013

Displays the unadjusted prevalence of patients who reported each health-related social need at least one time on a SNQ, followed by the absolute difference (ie, the difference in percentages between 2 groups, reported in percentage points) and the relative difference (ie, the percentage by which the transgender patients’ likelihood of each health-related social need differs relative to the cisgender patients’ likelihood). The absolute difference is included to represent the scale of the difference, and the relative difference is included to represent the proportional disparity. Source: Analysis of Montefiore's electronic health record data, 2018-2023.

Abbreviations: pp, percentage points; SNQ, Social Needs Questionnaire.

Table 4 presents the age-adjusted ORs for each HRSN among transgender patients compared with cisgender patients, using cisgender patients as the reference group. Several disparities were statistically significant, highlighting areas where transgender patients face notably higher odds of unmet social needs. A visual summary of these age-adjusted odds appears in Figure A1 as a forest plot (to access the appendix, please refer Supplementary material online).

**Table 4.** Age-adjusted odds for health-related social needs among transgender patients, with cisgender patients as reference.

Social need	aOR	SE	*P*-value	95% CI lower	95% CI upper	B–H critical value	Significant after correction
1. Housing instability	2.116	0.584	0.007	1.232	3.634	0.01	^a^
2. Poor-quality housing	2.108	0.584	0.007	1.224	3.629	0.015	^a^
3. Utilities shut off	1.511	0.56	0.265	0.731	3.126	0.045	
4. Food insecurity	1.554	0.432	0.112	0.902	2.679	0.035	
5. Healthcare transportation	1.529	0.479	0.174	0.828	2.824	0.03	
6. Healthcare costs	2.424	0.662	0.001	1.419	4.141	0.005	^a^
7. Child or elder care	1.159	0.564	0.761	0.447	3.009	0.05	
8. Legal help	2.026	0.765	0.062	0.966	4.248	0.025	
9. Interpersonal stress	1.284	0.501	0.522	0.598	2.758	0.04	
10. Interpersonal violence	3.384	1.801	0.022	1.192	9.605	0.02	

Displays the adjusted odds for each social need comparing odds among transgender patients, with cisgender patients as reference. All models adjusted for age. To correct for multiple comparisons, the B–H Procedure was applied. Differences in adjusted odds are considered significant if the *P*-value for each test is less than the B–H critical value, indicated by the letter “a” in the final column. Source: Analysis of Montefiore's electronic health record data, 2018-2023.

Abbreviations: aOR, adjusted odds ratio; B–H, Benjamini–Hochberg; CI, confidence interval; SE, standard error.

Transgender patients had more than twice the age-adjusted odds of reporting housing instability (aOR = 2.12, 95% confidence interval [CI] [1.23-3.63], *P* = 0.007), poor-quality housing (aOR = 2.11, 95% CI [1.22-3.63], *P* = 0.007), and high healthcare costs (aOR = 2.42, 95% CI [1.42-4.14], *P* = 0.001), all of which were significant after the B–H correction. Additionally, transgender patients had more than 3 times the age-adjusted odds of interpersonal violence (aOR = 3.38, 95% CI [1.19-9.61], *P* = 0.022). This result did not meet the B–H threshold, yet these high odds warrant further investigation.

In sensitivity analyses adjusting for age, insurance, and neighborhood, effect sizes were modestly attenuated but remained directionally consistent.

## Discussion

This study highlights disparities in HRSN screening, prevalence, and odds between transgender and cisgender patients at a safety-net hospital system. Results provide further evidence that transgender communities face heightened challenges, particularly in housing, healthcare costs, and interpersonal violence.^12^ These results were consistent in sensitivity analyses, suggesting insurance and neighborhood disadvantage partially, but not fully, account for the disparities observed. Findings align with broader research indicating that transgender people experience elevated rates of housing instability, healthcare cost burden, and other HRSNs including violence that critically affect health outcomes.^1,5,10^

This study adds to the sparse clinical research on HRSNs among transgender people. Screening varied by site, and transgender patients were screened less often despite greater unmet needs. Even with low overall screening, the pattern prompts an important question: if providers prioritize “high-risk” patients, why are transgender patients screened less?

### Housing

Transgender patients in this study had more than double the odds of experiencing housing instability and poor-quality housing, consistent with national evidence that transgender people, particularly women of color, are more likely to experience homelessness and precarious living conditions,^7,35^ as well as eviction and housing discrimination, even after adjusting for socioeconomic status.^36^ Some studies report lifetime homelessness rates among transgender people as high as 30%.^8^ In the current study, these patients were more than twice as likely to report poor-quality housing, concerns associated with environmental health-related illness such as asthma, hypertension, and other chronic diseases.^8^ Thus, poor-quality housing may be jeopardizing this community's health in myriad ways.^35^

Given our transgender cohort is relatively young, it is important to note that housing instability during early adulthood can disrupt education, employment, and social support systems, creating a cycle of socioeconomic and health instability that persists over the life course.^8^ Many transgender youth may leave unsafe or rejecting homes as a survival response, increasing their risk of homelessness and related harms.

At the policy level, these disparities highlight the need for targeted housing interventions, including enforcement of antidiscrimination laws, just-cause eviction protections, right-to-counsel programs, medical–housing partnerships, and tenant organizing.^37^ These strategies may help mitigate structural drivers of housing precarity. Given the national shortage of affordable housing, providers often struggle to connect patients to culturally appropriate resources,^38^ and clinicians can also contribute through broader policy advocacy.^39^

### Healthcare costs

In line with existing research indicating that transgender people face greater financial barriers to accessing both routine and gender-affirming healthcare,^9,16,17^ our transgender patients were more than twice as likely to report high healthcare costs. Transgender individuals frequently encounter health insurance coverage gaps, particularly for gender-affirming care, which may be excluded from insurance policies and/or criminalized by hostile legislation.^40^ The US Trans Discrimination Survey found that 33% avoided healthcare in the past year due to costs,^41^ which may contribute to delayed or foregone healthcare and thus increased risk for emergency room visits.^42^

In this study, transgender patients faced barriers to affordable healthcare even within a safety-net system. Few transgender patients in our sample had Medicare coverage, likely reflecting a younger cohort. However, national Medicare data suggest transgender enrollees are more often eligible through disability than age.^43^ In our safety-net setting, the small number of Medicare enrollees limits our ability to observe these national patterns. Findings highlight the importance of Medicaid expansion and nondiscrimination protections, such as Section 1557 of the ACA, to reduce barriers to access.^44^

### Violence

Though transgender patients had more than 3 times higher odds for interpersonal violence, this finding was no longer significant after correcting for multiple comparisons, likely due to the small sample size and wide CI (1.192, 9.605). However, this trend is consistent with national data that transgender people have 4 times higher risk for intimate partner violence, family abuse, police brutality, and forms of violence including public assaults.^10,11^ These myriad forms of violence, across structural and interpersonal contexts, cause cumulative harm.^45^ These trends underscore the need for routine screening and antiviolence interventions including legal aid, counseling, relocation support, and safe housing.^46,47^

### Implications for healthcare systems

Transgender patients were screened less often than cisgender patients, a reversal of the patterns seen for other marginalized groups in our sample. These findings underscore the need for healthcare systems to implement HRSN screening approaches that explicitly account for gender identity. Emerging federal and state efforts to standardize HRSN screening (eg, the Centers for Medicare & Medicaid Services (CMS) Accountable Health Communities (AHC) Health-Related Social Needs (HRSN) Screening Tool) should incorporate equity metrics across gender identities, so transgender patients are not excluded from quality reporting or referrals. Although universal screening may be considered ideal, some health systems still rely on targeted approaches in resource-constrained settings.^22^

However, while clinic staff may identify some groups as “high risk” and prioritize them for screening, this risk-based logic has been critiqued^23^ and appears to break down for transgender patients. Staff may not perceive transgender people as being at elevated risk, or they may be unaware of a patient's transgender identity due to incomplete EHR documentation, identity nondisclosure, or limited cultural competence.^31^ Transgender patients may be systematically overlooked, despite material hardship. This underscores the limitations of provider discretion and the need for equity-informed screening protocols that incorporate, but do not rely solely on, perceived risk.^6^

Gender disparities in HRSNs suggest that healthcare providers and staff should be trained to recognize the unique challenges faced by transgender patients and to use referral pathways to transgender-affirming housing, employment, and financial support services. Policymakers could incentivize transgender cultural competency training through licensure requirements, continuing education, and Medicaid value-based payment models.

Previous studies suggest that integrating community health workers or social workers into healthcare teams can significantly improve outcomes for patients with HRSNs.^34^ Expanding such services within safety-net systems could ultimately improve transgender patients” health outcomes. To effectively address HRSN, policy initiatives can invest in community health worker and social work programs that directly address HRSNs, as well as federal standards that incentivize equity in screening.

### Limitations and future directions

First, the reliance on retrospective EHR data may result in incomplete documentation of transgender identity, as gender identity is not always accurately captured in medical records. This could lead to misclassification of some patients as cisgender. National efforts to standardize SOGI data collection are critical for measuring and mitigating disparities.^31^ Second, the relatively small sample size of transgender patients who completed the SNQ limits the generalizability of our findings. Larger studies with more representative transgender cohorts are needed to confirm the trends observed, particularly regarding interpersonal violence.^11^ Investigating the intersection of HRSNs and disparities in mental health and substance use disorders may be critical for informing holistic, intersectional interventions. Results are also limited by self-report and misclassification bias, as patients may underreport HRSNs due to disclosure concerns, stigma, and perceived lack of benefits.

Our study included only patients who accessed outpatient care within the health system, likely underestimating HRSNs among those facing the greatest barriers to access. Transgender people for whom healthcare costs pose an extreme barrier may be missing, biasing estimates. Because both trans identity and perceived social status may influence the likelihood of being screened, our analytic sample is limited by selection bias. This introduces potential collider stratification bias, wherein restricting analysis to those screened may inflate or minimize associations between trans identity and HRSNs.

There may also be ascertainment bias from unmeasured clinic- or provider-level variation in SOGI documentation and HRSN screening practices, and we lacked structured site identifiers across 200+ sites to adjust for this. Future studies can isolate patient-level disparities from provider- or clinic-level effects, yet screening will ideally be comprehensive across sites.^27^

A final limitation involves the capture of race and ethnicity data, which were derived from the EHR and may not consistently reflect self-identification.^48^ While self-report is ideal, some entries were likely inferred or auto-populated. This introduces the possibility of misclassification and provider bias, particularly for multiracial patients. The same limitation applies to our top–down classification of transgender identity, and we encourage a more robust, patient-centered approach.

**Figure:**
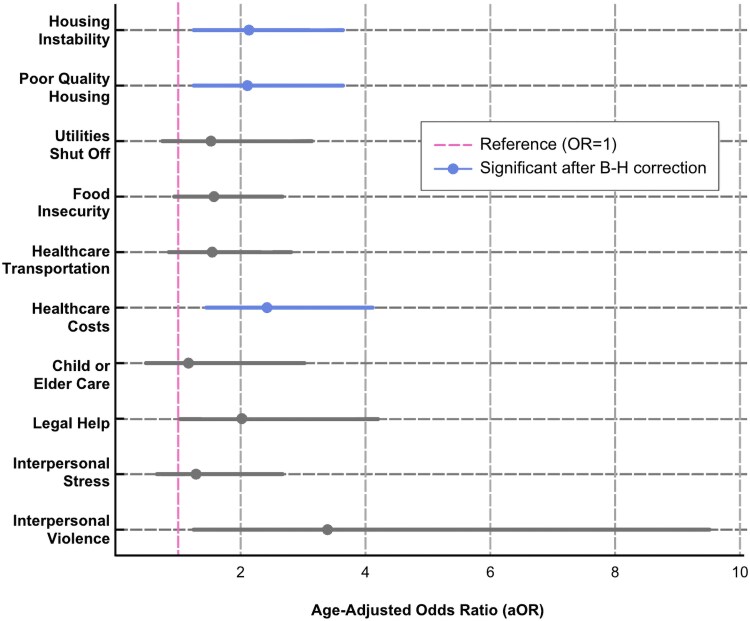


## Conclusion

This study highlights significant disparities in HRSNs between transgender and cisgender patients within a large safety-net healthcare system. Transgender patients face disproportionately high rates of housing instability, poor-quality housing, healthcare costs, and violence, which necessitate targeted interventions. Healthcare systems must prioritize the social and economic vulnerabilities of transgender people by implementing tailored screening and support. More radically, the proliferation of studies identifying disparities in HRSNs call for structural reorganization: for a reimagining of the material conditions of life to help transgender people survive and thrive.

## Supplementary Material

qxaf226_Supplementary_Data

## Data Availability

The data underlying this article cannot be shared publicly due to patient privacy protections and institutional restrictions on electronic health record data. The data will be shared on reasonable request to the corresponding author.
