# Supplementary material for: Fewer screens, greater needs: housing insecurity and healthcare costs for transgender patients in a safety-net system
Source: Health Aff Sch. 2025 Dec 5;4(1):qxaf226. doi: 10.1093/haschl/qxaf226 (PMC12849370; doi:10.1093/haschl/qxaf226)
Supplement: qxaf226_Supplementary_Data [file qxaf226_supplementary_data.zip › Breslow Manuscript Major Revision - Appendix (v2).docx]

**Appendix Material**

**Figure A1**

*Caption:* Forest plot of age-adjusted odds for health-related social needs among transgender patients compared with cisgender patients who completed the Social Needs Questionnaire.
 *Source/Notes:* SOURCE Authors’ analysis of Montefiore’s EHR data, 2018–2023. NOTES Horizontal lines represent 95% confidence intervals; the dashed line at OR = 1 indicates no difference between groups. OR = odds ratio; B-H = Benjamini-Hochberg method for multiple comparison adjustment; SNQ = Social Needs Questionnaire.

**Table A2**

*Caption:* Age-, insurance-, and neighborhood-adjusted odds for health-related social needs among transgender patients compared with cisgender patients who completed the Social Needs Questionnaire
*Source/Notes*: SOURCE Authors’ analysis of Montefiore’s EHR data, 2018–2023. NOTES This table displays adjusted odds for each social need among transgender patients, with cisgender patients as reference. Models adjust for age, insurance status [Medicaid, Medicare, Private (reference), Missing], and neighborhood characteristics [low-income, low-education, high-unemployment ZIP codes]. The Benjamini-Hochberg Procedure was applied to correct for multiple comparisons. Differences are considered significant if the p-value is less than the B-H critical value, indicated by an asterisk in the final column. aOR = adjusted odds ratio; S.E. = standard error; CI = confidence interval; B-H = Benjamini-Hochberg.

| TableA2. Age-, insurance-, and neighborhood-adjusted odds for health-related social needs among transgender patients | | | | | | | |
| --- | --- | --- | --- | --- | --- | --- | --- |
| **Social Need** | **aOR** | **S.E.** | **p-value** | **95% CI Lower** | **95% CI Upper** | **B-H Critical Value** | **Significant After Correction** |
| 1. Housing instability | 1.841 | 0.523 | 0.032 | 1.055 | 3.214 | 0.015 | * |
| 2. Poor quality housing | 1.982 | 0.555 | 0.014 | 1.145 | 3.431 | 0.010 | * |
| 3. Utilities shut off | 1.374 | 0.512 | 0.394 | 0.662 | 2.850 | 0.035 |  |
| 4. Food insecurity | 1.460 | 0.410 | 0.177 | 0.842 | 2.530 | 0.030 |  |
| 5. Healthcare transportation | 1.228 | 0.399 | 0.527 | 0.650 | 2.322 | 0.040 |  |
| 6. Healthcare costs | 2.408 | 0.666 | 0.001 | 1.401 | 4.141 | 0.005 | * |
| 7. Child or elder care | 1.029 | 0.501 | 0.953 | 0.396 | 2.670 | 0.050 |  |
| 8. Legal help | 1.970 | 0.753 | 0.076 | 0.931 | 4.167 | 0.025 |  |
| 9. Interpersonal stress | 1.294 | 0.508 | 0.511 | 0.600 | 2.793 | 0.045 |  |
| 10. Interpersonal violence | 3.094 | 1.656 | 0.035 | 1.084 | 8.831 | 0.020 | * |
| *Note*. This table displays the adjusted odds for each social need among transgender patients, with cisgender patients as reference. All models are adjusted for age, insurance status (Medicaid, Medicare, Private [reference], Missing), and neighborhood characteristics (i.e., whether patients lived in low-income, low education, and high-unemployment neighborhoods). To correct for multiple comparisons, the Benjamini-Hochberg Procedure was applied. Differences in adjusted odds are considered significant if the p-value for each test is < the B-H critical value, indicated by the * symbol in the final column. aOR, adjusted odds ratio; S.E., standard error; CI, confidence interval, B-H, Benjamini-Hochberg. | | | | | | | |
