# Supplementary figures and images for: Fewer screens, greater needs: housing insecurity and healthcare costs for transgender patients in a safety-net system
Source: Health Aff Sch. 2025 Dec 5;4(1):qxaf226. doi: 10.1093/haschl/qxaf226 (PMC12849370; doi:10.1093/haschl/qxaf226)

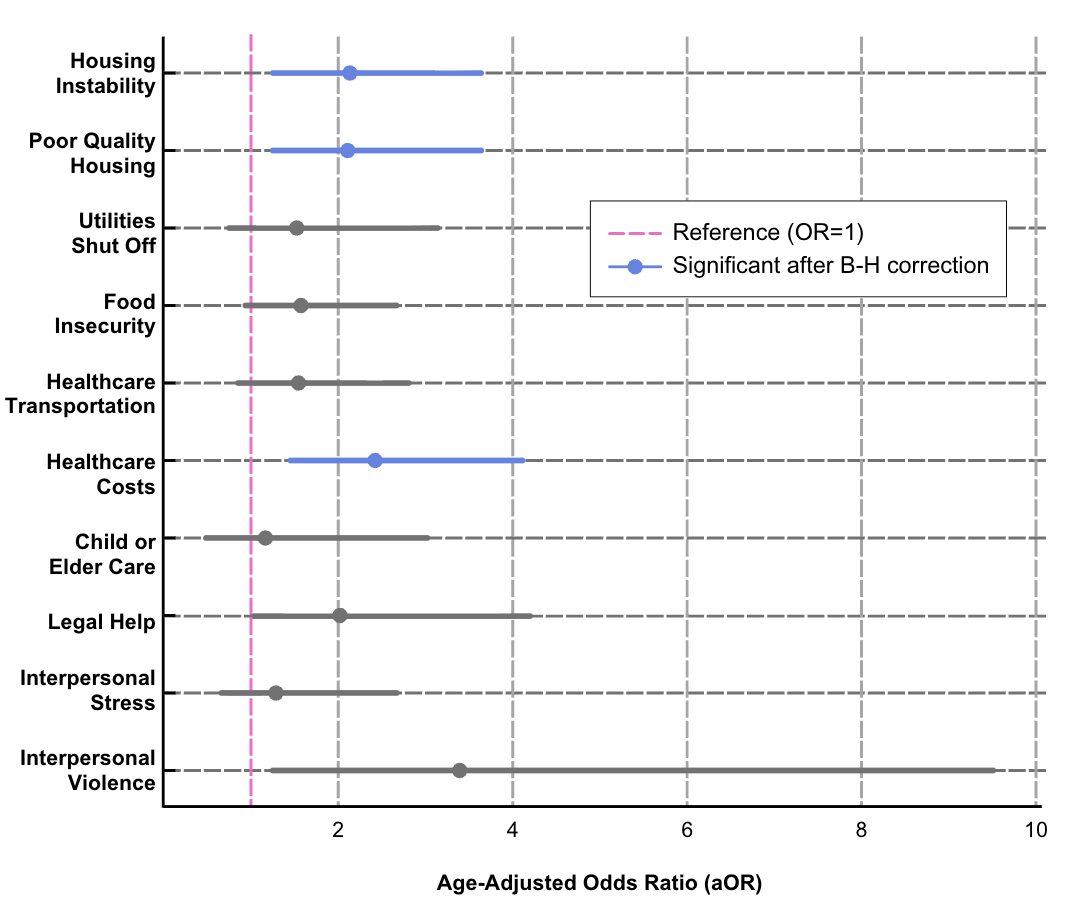

Supplement: qxaf226_Supplementary_Data [file qxaf226_supplementary_data.zip › Breslow Manuscript Major Revision - Figure.jpg]
